# Dynamic connectivity detection: an algorithm for determining functional connectivity change points in fMRI data

**DOI:** 10.3389/fnins.2015.00285

**Published:** 2015-09-04

**Authors:** Yuting Xu, Martin A. Lindquist

**Affiliations:** Department of Biostatistics, Johns Hopkins UniversityBaltimore, MD, USA

**Keywords:** functional connectivity, dynamic functional connectivity, resting state fMRI, change point detection, network dynamics

## Abstract

Recently there has been an increased interest in using fMRI data to study the dynamic nature of brain connectivity. In this setting, the activity in a set of regions of interest (ROIs) is often modeled using a multivariate Gaussian distribution, with a mean vector and covariance matrix that are allowed to vary as the experiment progresses, representing changing brain states. In this work, we introduce the Dynamic Connectivity Detection (DCD) algorithm, which is a data-driven technique to detect temporal change points in functional connectivity, and estimate a graph between ROIs for data within each segment defined by the change points. DCD builds upon the framework of the recently developed Dynamic Connectivity Regression (DCR) algorithm, which has proven efficient at detecting changes in connectivity for problems consisting of a small to medium (< 50) number of regions, but which runs into computational problems as the number of regions becomes large (>100). The newly proposed DCD method is faster, requires less user input, and is better able to handle high-dimensional data. It overcomes the shortcomings of DCR by adopting a simplified sparse matrix estimation approach and a different hypothesis testing procedure to determine change points. The application of DCD to simulated data, as well as fMRI data, illustrates the efficacy of the proposed method.

## 1. Introduction

Functional connectivity (FC) is the study of the temporal dependencies between distinct, possibly spatially remote, brain regions (Friston, 1994). Assessing FC using functional Magnetic Resonance Imaging (fMRI) data, has proven particularly useful for discovering patterns indicting how brain regions are related, and comparing these patterns across groups of subjects (Lindquist, 2008; Friston, 2011). In recent years, it has become one of the most active research areas in the neuroimaging community, and it is a central concept in the long term goal of understanding the human connectome (Sporns et al., 2005). The hope is that increased knowledge of networks and connections will help facilitate research into a number of common brain disorders.

FC is fundamentally a statistical concept, and is typically assessed using statistical measures such as correlation (Biswal et al., 1995), cross-coherence (Sun et al., 2004), and mutual information (Jeong et al., 2001). In the past few years it has become increasingly common to assume that the fMRI time series data follows a multivariate Gaussian distribution, and quantify FC using the estimated covariance, correlation or precision (inverse covariance) matrix (Varoquaux et al., 2010; Cribben et al., 2012, 2013). In this setting there is a well-known relationship between the estimated precision matrix and the underlying network graph of interest, and the use of algorithms for estimating sparse precision matrices (and thus graphs) have become critical (Friedman et al., 2008).

Most functional connectivity analyses performed to date have generally assumed that the relationship within functional networks is stationary across time. However, in recent years there has been an increased interest in studying dynamic changes in FC over time. These analyses have shown that rather than being static, functional networks appear to fluctuate on a time scale ranging from seconds to minutes (Chang and Glover, 2010). Here changes in both the strength and directionality of functional connections have been observed to vary across experimental runs (Hutchison et al., 2013), and it is believed that these changes may provide insight into the fundamental properties of brain networks.

When the precise timing and duration of state-related changes in FC are known before hand, it is possible to apply methods such as the psychophysiological interactions (PPI) technique (Friston et al., 1997) or statistical parametric networks analysis (Ginestet and Simmons, 2011). However, in many research settings the nature of the psychological processes being studied is unknown, particularly in resting-state fMRI (rfMRI), and it is therefore important to develop methods that can describe the dynamic behavior in connectivity without requiring prior knowledge of the experimental design. In the past couple of years, a number of such approaches have been suggested in the neuroimaging literature, including the use of sliding window correlations (Chang and Glover, 2010; Handwerker et al., 2012; Hutchison et al., 2013; Allen et al., 2014), change point models (Cribben et al., 2012, 2013), and volatility models (Lindquist et al., 2014).

**Figure 1:**
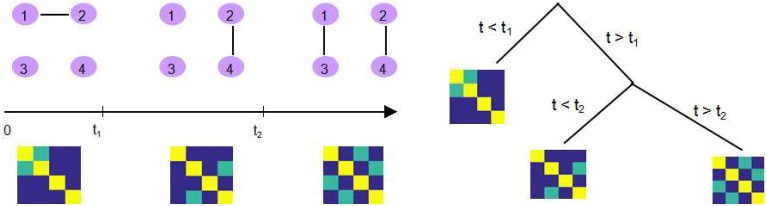
**An illustration of DCR**. **Left:** There exist two change points *t*_1_ and *t*_2_ where the connectivity between 4 ROIs changes as shown in the corresponding precision matrix. **Right:** DCR discovers the change points, recursively, using a binary search tree.

One example is dynamic connectivity regression (DCR), which is a data-driven technique for partitioning a time course into segments and estimating the different connectivity networks within each segment (Cribben et al., 2012). It applies a greedy search strategy to identify possible changes in FC using the Bayesian Information Criteria (BIC). While optimizing the BIC value within each subsequence, DCR utilizes the GLASSO algorithm to estimate a sparse inverse covariance matrix. This is followed by a secondary analysis of the candidate split points, where a permutation test is performed to decide whether or not the reduction in BIC at that time point is significant enough to be deemed a true change point. The structure of the DCR algorithm is briefly demonstrated in Figure 1.

While the DCR algorithm has proven useful for detecting changes in FC, it has two major drawbacks. First, the computational cost of the algorithm increases rapidly with the number of ROIs. As the number of ROIs surpasses 50, the computation time can become prohibitive. Second, DCR requires a number of user-specified input parameters, some of which may be difficult to optimize without in-depth knowledge of the experiment and familiarity with the algorithm.

In this work, we introduce the Dynamic Connectivity Detection (DCD) algorithm for change point detection in fMRI time series data, as well as the estimation of a graph representing connectivity within each partition. It builds upon the basic DCR framework, using the same binary search tree structure to recursively identify potential change points. However, it replaces a number of critical components of DCR, including the manner in which the sparse matrix estimation is performed and significant change points determined. An adaptive thresholding approach is used to estimate a sparse covariance matrix, which provides a significant speed up in computation time compared to the GLASSO algorithm, and improves scalability. In addition, the permutation test used to detect significant change points is replaced by an alternative hypothesis test. Because of these changes, all the input parameters in the DCD algorithm have a clear interpretation in the context of hypothesis testing, allowing users to specify the desired control of Type I and Type II errors.

This paper is organized as follows. In Section 2 we begin by briefly reviewing the basic steps of DCR, followed by a discussion of sparse parameter estimation, and a description of the new DCD algorithm for single-subject change point detection and graph estimation. Thereafter we demonstrate the performance of DCD in Sections 3 and 4 by applying the method to a series of simulation studies and experimental data. The obtained results are contrasted with similar results obtained using DCR. The paper concludes with a discussion.

## 2. Methods

Consider fMRI data from a single subject consisting of multivariate time series, where each dimension corresponds to activity from a single region of interest (ROI). Assume that the measurement vector at each time point follows a multivariate Gaussian distribution, whose parameters may vary across time. Throughout, we denote the measurement at time *t* as **y**(*t*) (1 ≤ *t* ≤*T*), which represents a *J*-dimensional Gaussian random vector whose distribution is N(**μ**(*t*), Σ(*t*)).

The goal of DCD is to detect temporal change points in functional connectivity and estimate a sparse connectivity graph for each segment, where the vertices are ROIs and the edges represent the relationship between ROIs. More specificity, we seek to partition the time series into several distinct segments, within which the data follows a multivariate Gaussian distribution with a different mean vector or covariance matrix from its neighboring segments. Further, for each segment we seek to estimate a graph representing connectivity between ROIs in the segment.

The DCR algorithm (Cribben et al., 2012, 2013) was previously developed to deal with the same problem. While, DCR has proven efficient at detecting changes in connectivity for problems consisting of a small to medium (< 50) number of regions, it runs into computational problems as the number of regions becomes large (>100). The proposed DCD algorithm seeks to circumvent these issues by updating how (i) the underlying mechanisms by which change points are determined, and (ii) network structures are identified. Before discussing DCD in detail, we begin by giving a brief overview of DCR and sparse parameter estimation.

### 2.1. Dynamic connectivity regression (DCR)

The original DCR algorithm (Cribben et al., 2012), dealt with detecting change points in a group of subjects, but here we concentrate on the single subject case (Cribben et al., 2013). DCR aims at detecting temporal change points in functional connectivity and estimating a graph of the conditional dependencies between ROIs, for data that falls between each pair of change points. The measured signal is modeled as a Gaussian random vector where each element represents the activity of one region. The partitions in DCR are found using a regression tree approach. It attempts to first identify a candidate change point using the Bayesian Information Criterion (BIC), and then perform a permutation test to decide whether it is significant. If a significant change points is found, the same procedure is recursively applied to search for more changes points by further splitting the subset; see Figure 1 for an illustration.

The required user specified input parameters for the algorithm are:

1. Δ: the minimum possible distance between adjacent changes points, chosen based on prior knowledge about the fMRI experiment.
2. λ − *list*: the full regularization path of tuning parameters λ required by GLASSO.
3. ξ: the mean block size of the stationary bootstrap.
4. α: the significance level for the permutation test.
5. *N*_*b*_: the number of bootstrap samples.

Suppose we have a *J*-dimensional time series **Y**: = {**y**(*t*)}_1≤*t*≤*T*_, where the **y**(*t*)′*s* are assumed to be independent identically distributed random variables which follow a multivariate Gaussian distribution. Here the mean vector can be estimated using the sample mean, and a sparse precision matrix can be estimated using the GLASSO technique (see next section for more detail). In order to choose the appropriate tuning parameter λ needed for GLASSO, the full regularization path λ − *list* is run, and the optimal value is selected based on the value that minimizes the BIC. Finally, the model is refit without regularization, but keeping the zero elements fixed, and the optimized baseline BIC for the original time series, *b*_0_, is recorded.

For all possible split points *t* (Δ ≤ *t* ≤ *T* − Δ), the same procedure is repeated, and the BIC score for the two subsequences $Y_{1}:={\left\{y\left(t^{\prime }\right)\right\}}_{1\leq t^{\prime }\leq t}$ and $Y_{2}:={\left\{y\left(t^{\prime }\right)\right\}}_{t+1\leq t^{\prime }\leq T}$, denoted *b*_1_(*t*) and *b*_2_(*t*), respectively, are computed. A time point *t*_0_ is chosen as a candidate change point, if it (i) produces the smallest combined BIC score *b*_1_(*t*_0_)+*b*_2_(*t*_0_) for all possible split points *t*, and (ii) the combined BIC score is smaller than the baseline *b*_0_. In the continuation we let δ_*b*_ = *b*_0_ − (*b*_1_(*t*_0_)+*b*_2_(*t*_0_)) represent the decrease in BIC at *t*_0_.

Because change points are defined by a decrease in BIC, a random permutation procedure is used to create a 100(1 − α)% confidence interval for BIC reduction at the candidate change point *t*_0_, to determine whether it should be deemed a significant change point. Using a stationary bootstrap procedure with mean block size ξ, permuted time series are repeatedly created. Each time course is partitioned at time *t*_0_ and the BIC reduction is computed as described above. The procedure is performed *N*_*b*_ times, thus allowing for the creation of a permutation distribution for the BIC reduction. If δ_*b*_ is more extreme than the (1−α) quantile of the permutation distribution, we conclude *t*_0_ is a significant change point. This procedure is recursively applied to each individual partition until no further split reduces the BIC score.

### 2.2. Sparse parameter estimation

The estimation of the covariance and precision matrix is a critical step in identifying candidate change points in the DCR algorithm. While the number of ROIs *J* is moderate, and the length of time series *T* is large, the sample covariance matrix *S* is a consistent estimator of the covariance matrix Σ. However, in high dimensional settings, when *J* is large compared to the sample size *T*, *S* has an infinite determinant, leading to divergence in the numerical algorithm. Thus, sparsity constraints are required to estimate the covariance, or precision matrix, consistently.

In this section we discuss two methods for performing sparse matrix estimation. While the original DCR method imposes sparsity on the precision matrix, the proposed DCD algorithm instead seeks to estimate a sparse covariance matrix. By making this shift, we can use a newly developed adaptive thresholding approach that provides a faster, more scalable solution to the change point problem described above. Statistically this changes the interpretation of the problem, as zeros in the precision matrix correspond to conditional independence between variables, while zeros in a covariance matrix correspond to marginal independence between variables. In a series of simulations and an application to real data we examine the implications of this choice.

#### 2.2.1. Graphical LASSO (GLASSO)

The Least Absolute Shrinkage and Selection Operator (LASSO) technique (Tibshirani, 1996), is often used for shrinkage and feature selection in regression problems. It adds an *L*_1_ penalty term to the objective function, thus producing more interpretable models with some coefficients forced to be exactly zero. The Graphical LASSO (GLASSO) (Friedman et al., 2008) is an extension of this idea to graphical models, aimed at estimating sparse precision matrices. Based on the assumption that the observed data vectors {**y**(*t*)}_1≤*t*≤*T*_ follow a multivariate Gaussian distribution with covariance matrix Σ, it adds an *L*_1_ norm penalty to the elements of the precision matrix Ω = Σ^−1^, and estimates the mean vector **μ** and precision matrix Ω by maximizing the penalized log-likelihood. After substituting the sample mean (the MLE of **μ**) into the objective function, this reduces to:

$$\log det(\Omega )-tr(S\Omega )-\lambda \|\Omega {\|}_{1}$$

where *S* is the empirical covariance matrix, and the parameter λ controls the amount of regularization. Maximizing the penalized profile log-likelihood gives a sparse estimate of Ω.

If the *ij*^*th*^ element of matrix Ω is zero, the variables *y*_*i*_(*t*) and *y*_*j*_(*t*) are conditionally independent, given the other variables. We can therefore define a connectivity graph *G* = (*V, E*) with the ROIs the vertices in *V*, and prune the edge between vertices *i* and *j* if the variables are conditionally independent. Thus, increasing the sparsity of Ω provides a sparser graphical representation of the relationship between the variables.

#### 2.2.2. Adaptive thresholding approach

Here we introduce an adaptive thresholding approach that allows one to estimate a sparse covariance matrix. Again, assume the data {**y**(*t*)}_1≤*t*≤*T*_ follows an i.i.d. multivariate Gaussian distribution N(**μ**, Σ). In this setting, the sample mean

$$\hat{\mu }=\frac{1}{T}\sum_{1\leq t\leq T}y(t)$$

is a consistent estimator of $\hat{\mu }$.

To estimate the covariance matrix, we begin by using the empirical covariance matrix

$$\hat{\Sigma }=\frac{1}{T}\sum_{1\leq t\leq T}(y(t)-\hat{\mu }{)}^{T}(y(t)-\hat{\mu })$$

as a candidate estimator of Σ. To achieve sparsity we investigate whether individual elements should be set equal to zero following an idea of Cai and Liu (2011), where a method to model the distribution of ${\hat{\Sigma }}_{ij}$ is proposed.

Let $X_{t}^{ij}:=\left(y_{i}\left(t\right)-\mu_{i}\right)\left(y_{j}\left(t\right)-\mu_{j}\right)$, where a subscript represents a single dimension of a vector, then the *ij*^*th*^ element of $\hat{\Sigma }$ is:

(1) $${\hat{\Sigma }}_{ij}=\frac{1}{T}\sum_{1\leq t\leq T}X_{t}^{ij}={\bar{X}}^{ij}$$

Now $X_{1}^{ij},X_{2}^{ij},\ldots X_{T}^{ij}$ is a sequence of i.i.d. random variables with $E\left[X_{t}^{ij}\right]=E\left[\left(y_{i}\left(t\right)-\mu_{i}\right)\left(y_{j}\left(t\right)-\mu_{j}\right)\right]=\Sigma_{ij}$ by definition, and further assume $Var\left[X_{t}^{ij}\right]=\delta_{ij}^{2}<\infty$. Then by the Central Limit Theorem,

$$\sqrt{T}({\hat{\Sigma }}_{ij}-\Sigma_{ij})\to N(0,\delta_{ij}^{2})$$

A natural estimate of $\delta_{ij}^{2}$ is given by:

(2) $$\hat{\delta_{ij}^{2}}=\frac{1}{T}\sum_{1\leq t\leq T}{\left(X_{t}^{ij}-{\bar{X}}^{ij}\right)}^{2}$$

Alternatively, one can use the Jackknife technique to estimate the variance of estimator ${\hat{\Sigma }}_{ij}$ directly (see Appendix B).

Using this result, we can test *H*_0_ : Σ_*ij*_ = 0 *vs*. *H*_1_ : Σ_*ij*_ ≠ 0 at significance level η as follows:

$$|\frac{\sqrt{T}{\hat{\Sigma }}_{ij}}{\hat{\delta_{ij}}}|=\frac{T|{\hat{\Sigma }}_{ij}|}{\sqrt{\sum_{t=1}^{T}{\left(X_{t}^{ij}-{\bar{X}}^{ij}\right)}^{2}}}>z_{1-\eta /2}$$

If we successfully reject the null hypothesis, we can conclude that Σ_*ij*_ ≠ 0 and keep ${\hat{\Sigma }}_{ij}$ as the estimator for Σ_*ij*_. Otherwise we modify the candidate estimator and set ${\hat{\Sigma }}_{ij}=0$. Similarly, using the diagonal elements of $\hat{\Sigma }$ as estimates of the variance of $\hat{\mu }$, we can perform a hypothesis testing for each element of **μ** and obtain a sparse estimate of $\hat{\mu }$. Since the testing procedure is performed for a potentially large number of parameters, we need to correct for multiple comparisons (Lindquist and Mejia, 2015).

### 2.3. Dynamic connectivity detection (DCD)

The DCD algorithm seeks to speed up the DCR algorithm, while achieving equivalent, or improved, results. The general procedure of DCD is similar to DCR, where a candidate split point is identified based on whether it further maximizes a likelihood-based function, and a hypothesis test is performed to decide whether this candidate split point is statistically significant. If a significant change point is found, the procedure is applied recursively to each of the two subsequences in order to find further split points.

**Figure 2:**
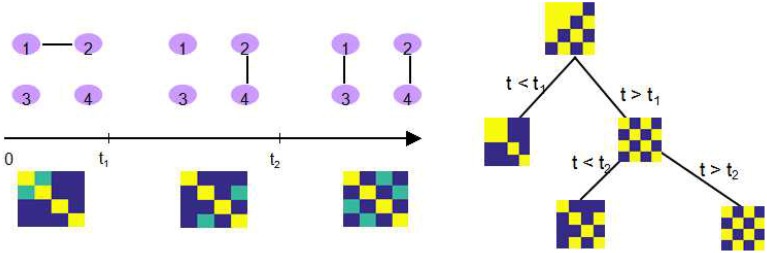
**An illustration of how the sparsity structure is recorded in DCD**. **Left:** The split points *t*_1_ and *t*_2_, and the corresponding covariance matrix within each partition. Yellow elements in the covariance matrix plot represents 1, green elements 0.5, and blue elements 0. **Right:** DCD uses a binary mask to record the sparsity structure at each node of the binary search tree.

The major improvement from DCR to DCD is that we incorporate the adaptive thresholding approach as our sparse matrix estimation method, which successfully improves upon the computational efficiency. In addition, during each step, a binary “mask” representing the non-zero parameter elements (in the mean vector and covariance matrix) is saved for each partition. If an additional change point is found for this partition, the “mask” is imposed on the parameters of both “child” partitions (the two subsets of time series created by splitting the data at the change point). This implies that if the estimate of one element of the covariance matrix for some partition is zero, then the estimate of corresponding element in any sub-partition will also be zero. The recursive sparsity feature is illustrated in Figure 2.

All input parameters in DCD have a clear statistical interpretation, enhancing its user-friendliness. The required user specified input parameters for the algorithm are:

1. α: the type I error bounds for the hypothesis tests used to determine significant splits.
2. β: the type II error bounds for the hypothesis test used to determine significant splits.
3. η: the type I error bound for the hypothesis test used to determine the sparsity of the covariance matrix.

Since the length of the time series partition affects statistical inference, we need to calculate the minimum partition length Δ needed to achieve the desired error bounds. We apply a power analysis based on a two sample *t*-test to calculate Δ from the inputs α and β; for details please refer to Appendix A.

Given a *J*-dimensional time series **Y**: = {**y**(*t*)}_1≤*t*≤*T*_, we begin by calculating the maximized baseline log-likelihood *L*_0_ under the assumption that

$$y(t)\;\overset{i.i.d}{\sim }\;N(\mu_{0},\Sigma_{0}),\;1\leq t\leq T.$$

Hence, the log-likelihood function is given by

(3) $$\begin{array}{l} L(\mu_{0},\Sigma_{0}|Y)\propto -\sum_{t\;=\;1}^{T}(y(t)-\mu_{0}{)}^{T}\Sigma_{0}^{-1}(y(t)-\mu_{0})\\ \;-T\log(\det\Sigma_{0}). \end{array}$$

We first calculate the sample mean and sample covariance matrix as the maximum likelihood estimator of **μ**_0_ and Σ_0_, and then further improve the estimator by performing the adaptive thresholding method described in Section 2.2.2, in order to obtain a sparse mean vector ${\hat{\mu }}_{0}$ and sparse covariance matrix ${\hat{\Sigma }}_{0}$.

The maximized log-likelihood function can now be expressed as:

$$L_{0}=-T(tr({\hat{\Sigma }}_{0}^{-1}S)+\log(\det{\hat{\Sigma }}_{0}))$$

where *S* is the normalized scatter matrix:

$$S=\frac{1}{T}\sum_{1\leq t\leq T}(y(t)-{\hat{\mu }}_{0}{)}^{T}(y(t)-{\hat{\mu }}_{0})$$

While calculating the sparse structure of parameter **θ**_0_ = (**μ**_0_, *vec*{Σ_0_}), a binary array *mask* is saved, indicating the non-zero elements of θ_0_. It is assumed that any subsequence of the time series will satisfy the parent sparsity property.

For any possible candidate split point *t* (Δ ≤ *t* ≤ *T* − Δ), assume the two subsequences $Y_{1}:={\left\{y\left(t^{\prime }\right)\right\}}_{1\leq t^{\prime }\leq t}$ and $Y_{2}:={\left\{y\left(t^{\prime }\right)\right\}}_{t+1\leq t^{\prime }\leq T}$ follow multivariate Gaussian distribution with parameters **θ**_1*t*_ = (**μ**_1*t*_, *vec*{Σ_1*t*_}) and **θ**_2*t*_ = (**μ**_2*t*_, *vec*{Σ_2*t*_}), respectively. Here only the upper triangular elements are used when vectorizing the covariance matrix. The dimension of the parameter vector is therefore *J* + *J*∗(*J* + 1)/2 = (*J* + 1)(*J* + 2)/2. Next, the maximum likelihood estimators ${\hat{\theta }}_{it}^{ML}$ (*i* = 1,2) are computed, imposing the parent sparsity structure by taking the Hadamard product with the *mask* vector:

$${\hat{\theta }}_{it}={\hat{\theta }}_{it}^{ML}⊗mask,\;i=1,2$$

Now the maximized log-likelihood under current split point *t* can be obtained as follows:

$$L_{t}=L({\hat{\theta }}_{1t}|Y_{1})+L({\hat{\theta }}_{2t}|Y_{2}).$$

Similar to DCR we can now step through all possible candidate split points and find the one, denoted *t*_0_, which shows the maximum improvement in log-likelihood *L*_*t*_ compared to *L*_0_:

$$t_{0}=\underset{t}{argmax}{\left(L_{t}-L_{0}\right)}_{+}$$

If the maximum *L*_*t*_0__ is less than the baseline *L*_0_, the DCD procedure returns no detected split points; otherwise a set of hypothesis tests are performed to determine whether *t*_0_ is a significant change point.

For the sake of clarity, denote the Gaussian distribution parameters of the two subsequences as **θ**_*i*_ = (**μ**_*i*_, *vec*{Σ_*i*_}): = **θ**_*it*_, (*i* = 1,2). We now seek to test:

(4) $$\begin{array}{l} H_{j0}:\theta_{1}(j)=\theta_{2}(j)\;vs.\;H_{j1}:\theta_{1}(j)\neq \theta_{2}(j),\\ \;j\in \{j^{\prime }:mask(j^{\prime })=1\} \end{array}$$

If any of the non-zero parameters are significantly different for the two subsequences, i.e., if we reject any of the null hypotheses, then we conclude that *t*_0_ is a significant change point for partitioning the time series **Y**. We use Bonferroni correction to control the family-wise error rate (FWER), and reject *H*_*j*0_ if the *p*-value is less than $\frac{\alpha }{\underset{j^{\prime }}{\sum }mask\left(j^{\prime }\right)}$.

To perform each test we use Welch's *t*-test (two-sample *t*-test for unequal variance). For *j* ≤ *J*, use the diagonal element of $\hat{\Sigma }$ as an estimate of the variance of $\hat{\mu }$; and for *j* > *J*, use the estimator described in Equation (2) to estimate the variance of each element of $\hat{\Sigma }$. If *t*_0_ is identified as a significant change point, continue searching for more change points by recursively repeating the above procedure on the two “child” subsequences until no further change points are returned; otherwise finish the DCD procedure by returning a null value.

The complete procedure for performing the DCD algorithm is summarized below:

1. Take the input parameters α, β, η, and calculate the minimum partition length Δ as described in Appendix A.
2. Consider the full multivariate time series with length *T*, calculate the sparsity structure of its multivariate normal distribution parameters as described in Section 2.2.2, estimate the mean vector and a sparse covariance matrix accordingly, and calculate the baseline likelihood function *L*_0_.
3. For each value of t ranging from Δ to *T* − Δ, partition the time series into two subsequences {1 : *t*} and {*t* + 1 : *T*}, calculate the sparsity structure of parameters based on the parent sparsity structure from Step (2), then calculate the combined likelihood function using the estimated sparse parameters.
4. Find the time point which produces the largest increase in combined likelihood function, perform the hypothesis test described in Equation (4) to determine whether it is a significant change point. If yes, split the time series into two partitions accordingly.
5. Apply Steps (2–4) recursively to each partition until no further change points are found.
6. After detecting all change points, estimate a connectivity graph for each partition using a sparse matrix estimation technique, such as Adaptive Thresholding Approach to obtain a covariance graph or GLASSO to obtain a connectivity graph.

## 3. Simulations

A series of simulations were performed to test the efficacy of the new DCD algorithm, and compare its performance to the DCR method. For this reason, we adopt simulation settings inspired by those found in the original DCR work (Cribben et al., 2012). However, in contrast to that work, for each simulation the connectivity pattern and strength between nodes remains the same across different subjects, since our focus is on the single subject case instead of on group-level inference. In addition, the object of each simulation in this paper is focused on identifying the timing of the connectivity change points, rather than explicitly assessing the quality of the estimation of the underlying graphs.

**Figure 3:**
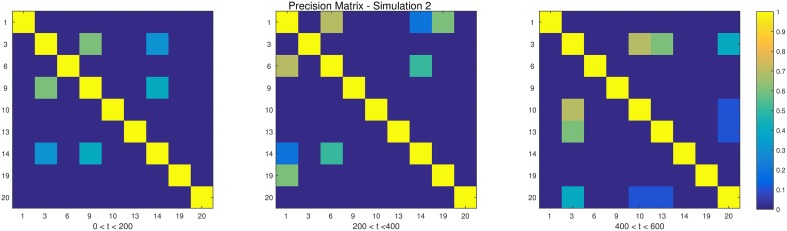
**The dependency structure used in each of the three partitions of Simulation 2**.

**Figure 4:**
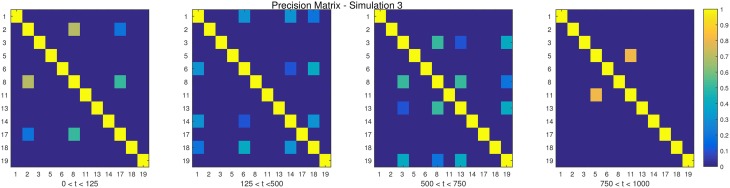
**The dependency structure used in each of the four partitions of Simulation 3**.

**Figure 5:**
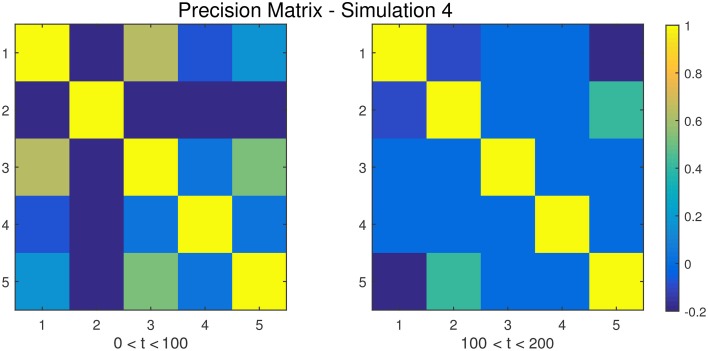
**The dependency structure between regions 1–5 (all other regions are conditionally independent) used in each of the two partitions of Simulation 4**.

**Figure 6:**
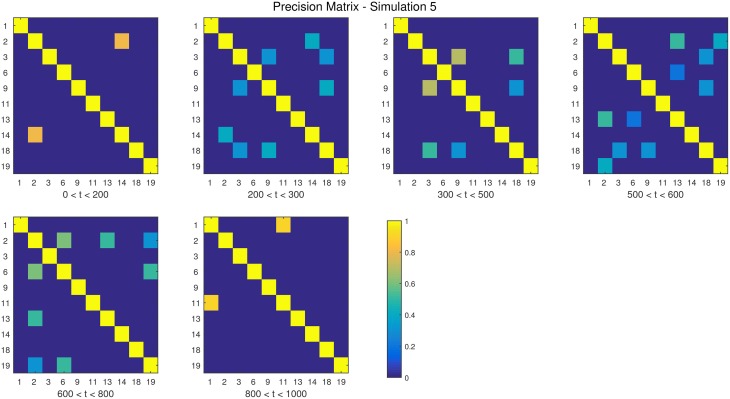
**The dependency structure used in each of the six partitions of Simulation 5**.

**Figure 7:**
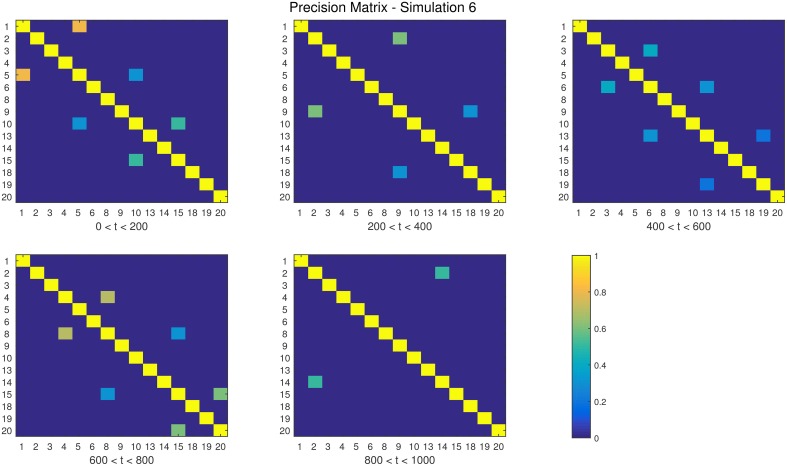
**The dependency structure used in each of the five partitions of Simulation 6**.

The descriptions and parameter settings for each simulation are listed below. Here *N*, *T*, and *p* represent the number of subjects, the length of the time series, and the number of regions, respectively. The true dependency between ROIs (i.e., the precision matrices) are shown as heat maps in Figures 3–7. More details regarding the exact strength of these connections can be found in Appendix C. Here the notation (*i, j*) = *k* indicates that the (*i, j*) element of the precision matrix takes the value *k*. All unspecified diagonal elements are one and non-diagonal elements are zero. In the latter case, the ROIs were made up of i.i.d. Gaussian noise indicating a lack of functional connectivity. Hence, each simulation is created assuming sparsity in the precision matrix, which should theoretically benefit DCR over DCD, which imposes sparsity in the covariance matrix.

For each simulation, both the DCD and DCR approaches were applied to the *N* subjects individually. Since the DCR algorithm has many parameters, and according to previous work several are insensitive to change, we fix several of them as follows:

$$\Delta =50,\;\lambda -list=(2^{0},2^{-1},...,2^{-9}),\;N_{b}=50,\;\xi =\Delta /2.$$

APP For DCD, we fix η = 0.05. All remaining parameters are altered depending on the simulation setting.

Below we list a brief description of each simulation study.

- **Simulation 1**
  *Description:* The data is white noise with no connectivity change points.
  *Size: N* = 20, *T* = 1000, *p* = 20
  *DCD parameters:* (α, β) = (0.05, 0.1);
  *DCR parameters:* α = 0.05.
- **Simulation 2**
  *Description:* There are two change points at times 200 and 400. Spikes are imposed onto the time series, imitating a common artifact found in fMRI data. For each subject there are 5 randomly placed spikes, each with magnitude 15.
  *Size: N* = 20, *T* = 1000, *p* = 20
  *DCD parameters:* (α, β) = (0.05, 0.1);
  *DCR parameters:* α = 0.05.
- **Simulation 3**
  *Description:* There are three change points at times 125, 500, and 750.
  *Size: N* = 15, *T* = 1000, *p* = 20
  *DCD parameters:* (α, β) = (0.05, 0.05);
  *DCR parameters:* α = 0.05.
- **Simulation 4**
  *Description:* There is a single change point at time 100.
  *Size: N* = 25, *T* = 200, *p* = 5
  *DCD parameters:* (α, β) = (0.05, 0.1);
  *DCR parameters:* α = 0.05.
- **Simulation 5**
  *Description:* There are five change points at times 200, 300, 500, 600, and 800.
  *Size: N* = 20, *T* = 1000, *p* = 20
  *DCD parameters:* (α, β) = (0.05, 0.05);
  *DCR parameters:* α = 0.05.
- **Simulation 6**
  *Description:* There are four change points at times 200, 400, 600, and 800.
  *Size: N* = 20, *T* = 1000, *p* = 20
  *DCD parameters:* (α, β) = (0.05, 0.05);
  *DCR parameters:* α = 0.05.

**Figure 8:**
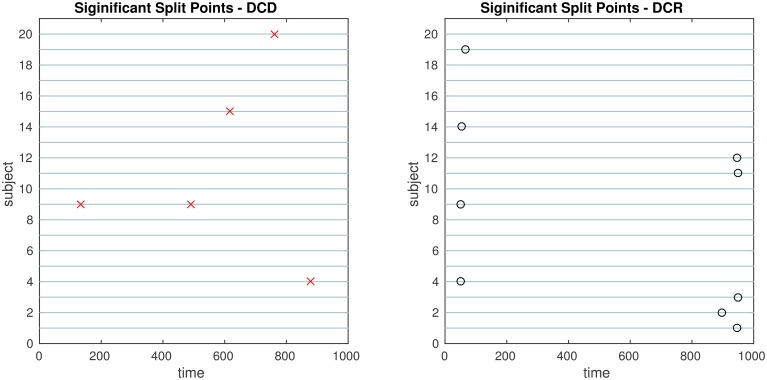
**The results of Simulation 1**. **Left:** The red crosses show significant split points found by DCD. **Right:** The blue circles show significant split points found by DCR. Here there should ideally be no change points for any of the subjects.

**Figure 9:**
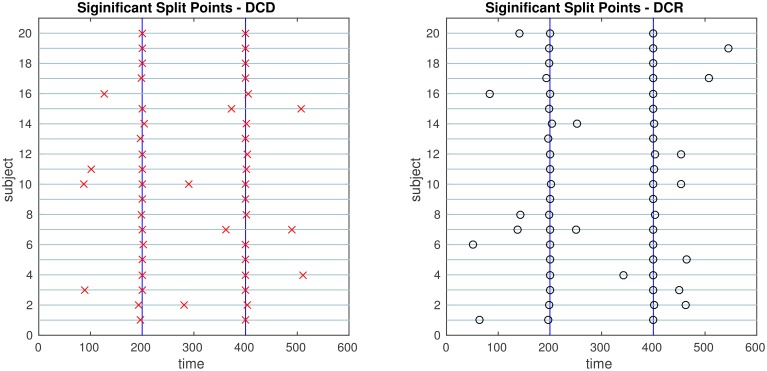
**The results of Simulation 2**. **Left:** The red crosses show significant split points found by DCD. **Right:** The blue circles show significant split points found by DCR. The blue vertical lines indicate the timing of the true change points.

**Figure 10:**
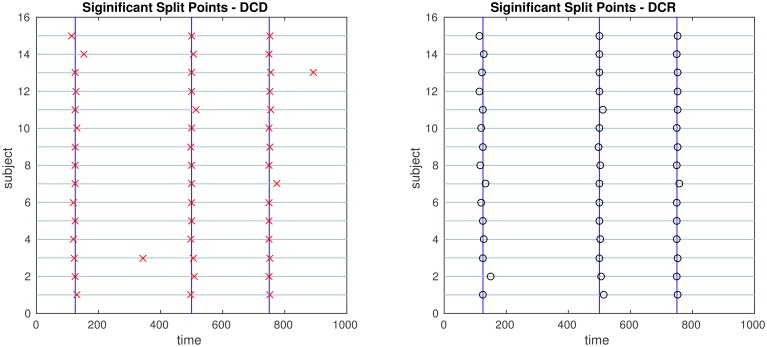
**The results of Simulation 3**. **Left:** The red crosses show significant split points found by DCD. **Right:** The blue circles show significant split points found by DCR. The blue vertical lines indicate the timing of the true change points.

**Figure 11:**
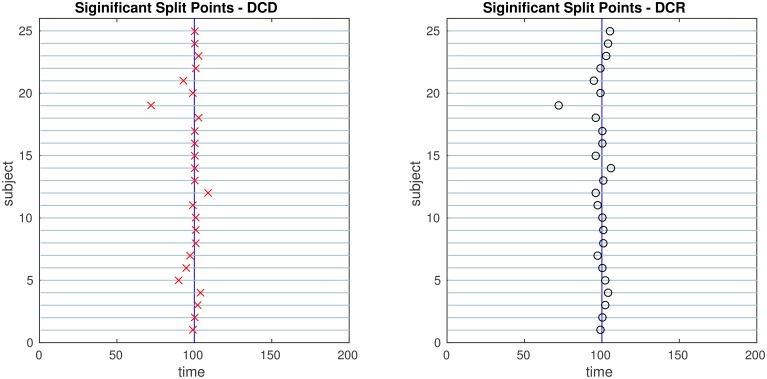
**The results of Simulation 4**. **Left:** The red crosses show significant split points found by DCD. **Right:** The blue circles show significant split points found by DCR. The blue vertical line indicates the true change points.

**Figure 12:**
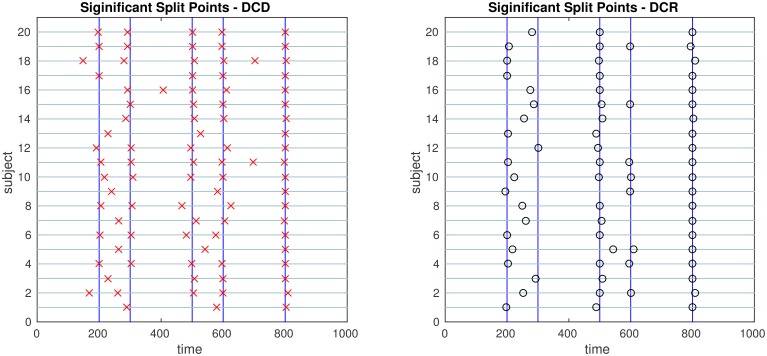
**The results of Simulation 5**. **Left:** The red crosses show significant split points found by DCD. **Right:** The blue circles show significant split points found by DCR. The blue vertical lines indicate the timing of the true change points.

**Figure 13:**
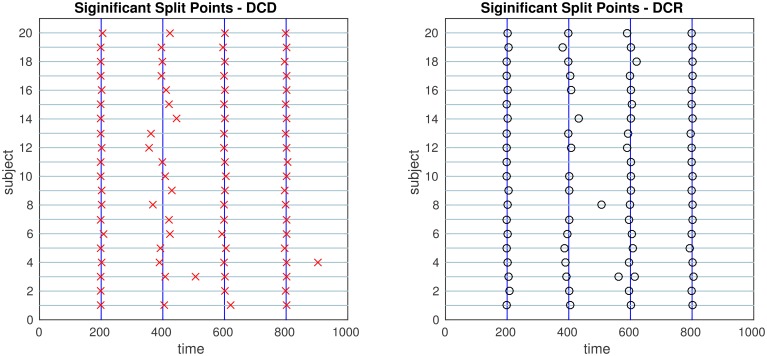
**The results of Simulation 6**. **Left:** The red crosses show significant split points found by DCD. **Right:** The blue circles show significant split points found by DCR. The blue vertical lines indicate the timing of the true change points.

The results of the simulations are shown in Figures 8–13. In each figure, the y-axis represents the subject number, while the x-axis represents time points. All red crosses in the left sub figures represent change points detected for each subject by DCD, and the blue circles are those detected by DCR. The blue vertical line indicates the true change points for each simulation setting. In Table 1, we list the respective runtimes of DCD and DCR for each simulation. The computing platform used was an Intel Core i5-3210M CPU 2.5 GHz with 16.0 GB RAM.

**Table 1:** **Runtime comparison between the DCD and DCR algorithms for each simulation**.

	**Simulation 1**	**Simulation 2**	**Simulation 3**	**Simulation 4**	**Simulation 5**	**Simulation 6**
DCR run time	249.163458	351.531306	408.477891	13.262008	585.510362	581.070222
DCD run time	8.005733	5.756054	12.363800	0.712059	16.958910	18.126004
ratio = $\frac{DCRtime}{DCDtime}$	31.1231	61.0716	33.0382	18.6249	34.5252	32.0573

Runtime is measured in units of seconds.

The results of Simulation 1, where there are no true change points, are shown in Figure 8. The DCD algorithm finds 5 false positive change points, whereas the DCR algorithm finds 9. Interestingly, the DCR false positives are primarily grouped at the time points Δ and *T* − Δ. The reason for this is that when adding the BIC score from two sub-series of lengths *n*_1_ and *n*_2_, where *n*_1_ + *n*_2_ = *n*, and assuming the number of parameters *k*_1_ ≈ *k*_2_ ≈ *k*, the total penalty term is *klog*(*n*_1_) + *klog*(*n*_2_) ∝ *log*(*n*_1_(*n*−*n*_1_)), which favors small or large values of *n*_1_ when minimizing the BIC. In addition, the runtime of DCD is approximately 30 times faster than DCR, providing a significant decrease in computation time.

The results of Simulation 2 are shown in Figure 9. Here there exist two true change points, the first at time 200, and the second at time 400. In addition, there are 5 spikes placed at random time points for each subject. Both algorithms do a good job of detecting the true change points in most cases, with a few instances of false positives for each. Here DCD is approximately 60 times faster than DCR in obtaining the results.

The results of Simulation 3 are shown in Figure 10. Here there exist three true change points, the first at time 125, the second at time 500, and the third at time 750. Clearly, both algorithms do an excellent job of detecting the true change points. Here DCD is approximately 30 times faster than DCR in obtaining the results.

Figure 11 shows the results of Simulation 4. Again, both algorithms do an excellent job of detecting the true change point, which is located at time 100, but DCD does so with a 20-fold increase in speed.

Finally, the results of Simulations 5 and 6 are shown in Figures 12, 13, respectively. In both cases the algorithms do an excellent job of detecting the true change points. However, DCD does so with a 30-fold increase in speed in both cases.

Although the main goal of DCD is to detect change points, and the estimation of a connectivity graph seems a byproduct, the accuracy of the covariance matrix or precision matrix estimation leads to better change point detection, and vice versa. Using the Adaptive Thresholding Approach, we need to control the family-wise error rate or false discovery rate. The estimation of a *J*-dimensional covariance matrix requires *O*(*J*^2^) hypothesis tests. In our simulation examples, we adjust the significance level η by η/*J*, to guard against being as conservative as Bonferroni correction, while still obtaining adequate control over the family-wise error rate. Results show that the estimation of the sparsity structure is accurate in most simulations. The list of the average proportion of correctly identified zero/non-zero elements of the covariance matrices are listed in Table 2.

**Table 2:** **Sparsity control results of covariance matrices**.

**Simulation**	**1**	**2**	**3**	**4**	**5**	**6**
Correct zero rate	0.9497	0.9568	0.9474	0.9529	0.4955	0.9461
Correct non-zero rate(TP)	1	0.9763	0.9644	0.6535	0.9837	0.9628
False positive rate (average)	0.0503	0.0432	0.0526	0.0471	0.5045	0.0539

In summary, in each of the “low dimensional” simulations described above, with the number of ROIs ~ 20, DCR achieves similar results as DCD with a significant speed-up in runtime. However, to investigate how well the methods scale to a more “high dimensional” settings, we expand upon two of the simulations to inspect how computational time changes as a function of the number of ROIs for the two algorithms.

In the first (denoted 2B), we generated 80 ROIs data for 50 subjects under the same settings as described in Simulation 2. Here only the first 20 nodes contain information, and the remaining are simply white noise. We ran DCD and DCR using ROIs 1:*r*, where *r* ranged from 20 to 80 in increments of 5. In the second (denoted 4B), we generated 70 ROIs for 50 subjects under the same settings as described in Simulation 4. Here only the first 5 nodes contain information, while all remaining nodes are white noise. We ran DCD and DCR on a subset of ROIs numbered 1:*r*, where *r* ranged from 5 to 70 in increments of 5.

**Figure 14:**
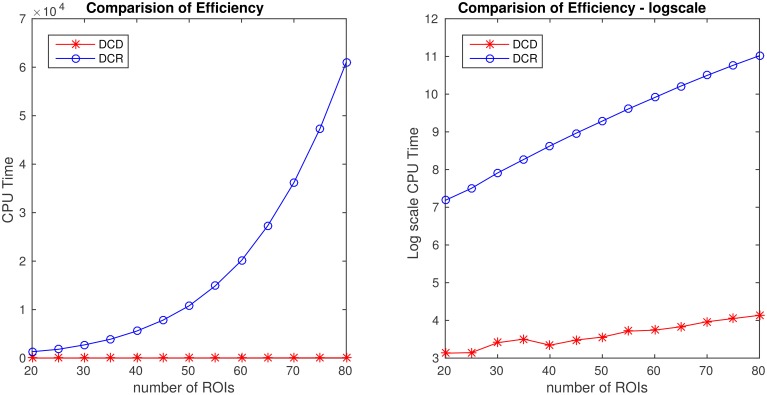
**Runtime for Simulation 2B as a function of number of nodes for both DCD and DCR on both regular (left) and log-scale (right)**. Clearly, DCD scales much better than DCR.

**Figure 15:**
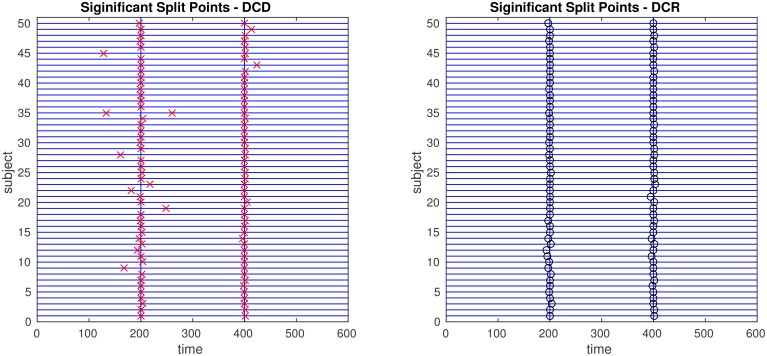
**The results of Simulation 2B**. **Left:** The red crosses show significant split points found by DCD. **Right:** The blue circles show significant split points found by DCR. The blue vertical lines indicate the timing of the true change points.

The results of Simulation 2B are summarized in Figures 14, 15. From Figure 14 it is clear that the computation time for DCR increases exponentially with the number of ROIs, while the computation time for DCD is much shorter and nearly linear. Though the results of DCR appear slightly better than DCD (see Figure 15), with less deviations from the true change points, this comes at a substantial computational cost.

**Figure 16:**
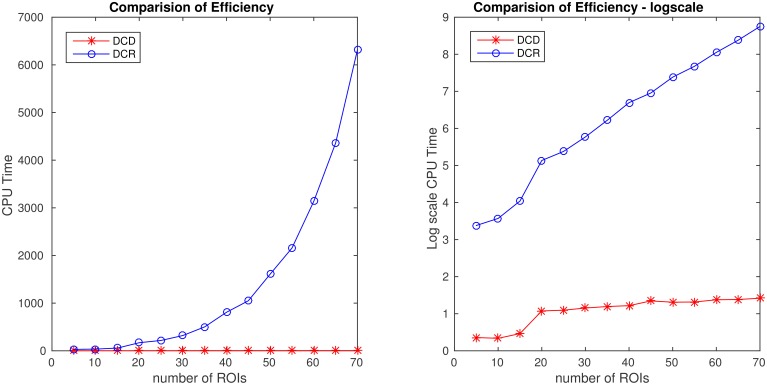
**Runtime for Simulation 4B as a function of number of nodes for both DCD and DCR on both regular (left) and log-scale (right)**. Clearly, DCD scales much better than DCR.

**Figure 17:**
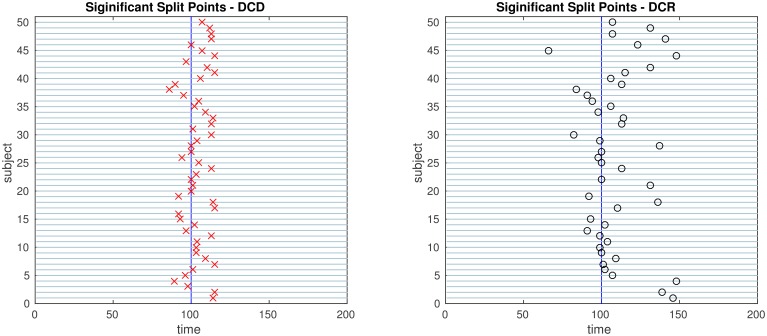
**The results of Simulation 4B**. **Left:** The red crosses show significant split points found by DCD. **Right:** The blue circles show significant split points found by DCR. The blue vertical line indicates the true change point.

The results of Simulation 4B are summarized in Figures 16, 17. Based on Figure 16 it is clear that the computation time for DCR increases exponentially with the number of ROIs, while the computation time for DCD is much shorter and has a near linear increase. In addition, judging by Figure 17 the algorithm also appears to more accurately detect the timing of the true change points.

## 4. Application to experimental data

### 4.1. Social evaluative threat experiment

The data was taken from an experiment where subjects performed an anxiety-inducing task while fMRI data was acquired (Wager et al., 2009). This is the same data set used in the previous DCR papers (Cribben et al., 2012, 2013), as well as in other papers exploring mean change points (Lindquist et al., 2007; Robinson et al., 2010). The task was a variant of a well-studied laboratory paradigm for eliciting social threat, during which participants were asked to give a speech under evaluative pressure. It consisted of an off-on-off design, with an anxiety-provoking speech preparation task sandwiched between two lower-anxiety rest periods. Prior to the scanning session, subjects were informed that they were to be given 2 min to prepare a 7 min speech, the topic of which would be revealed to them during scanning, that would be delivered to a panel of expert judges after the scanning session. However, they were told that there was a small chance that they would be randomly selected not to give the speech. After the start of fMRI acquisition, during the initial 2 min resting period subjects viewed a fixation cross. At the end of this period, an instruction slide appeared describing the speech topic for 15 s (“why you are a good friend”). The slide instructed subjects to prepare enough for the entire 7 min period. After 2 min of silent preparation, a second instruction screen appeared for 15 s that informed subjects that they would not have to give the speech. The functional run concluded with an additional 2 min period of resting baseline.

During the course of the experiment a series of 215 functional images were acquired (TR = 2 s). A detailed description of the data acquisition and preprocessing can be found in previous work (Wager et al., 2009). In order to create ROIs, time series of voxels were averaged across pre-specified regions of interest. We used data consisting of 4 ROIs and heart rate for 23 subjects. The 4 ROIs were chosen due to the fact that they showed a significant relationship to heart rate in an independent data set. They included the ventral medial prefrontal cortex (VMPFC), the anterior medial prefrontal cortex (mPFC), the striatum/pallidum, and the dorsal lateral prefrontal cortex (DLPFC)/inferior frontal junction (IFJ). The temporal resolution of the heart rate was 1 s compared to 2 s for fMRI data, so it was down-sampled by taking every other measurement.

Both the DCD and DCR approaches were applied to the 23 subjects individually. For the DCD algorithm, we used (α, β, η) = (0.1, 0.1, 0.05) as input parameters, and the runtime was 0.92 s. For the DCR algorithm, we adopted similar parameter settings used in Cribben et al. (2013), where we used the following settings: Δ = 40, λ − *list* = (1, 2^−1^, …, 2^−9^), α = 0.1, *N*_*b*_ = 50, and ξ = 20. The runtime for DCR was 32.14 s.

**Figure 18:**
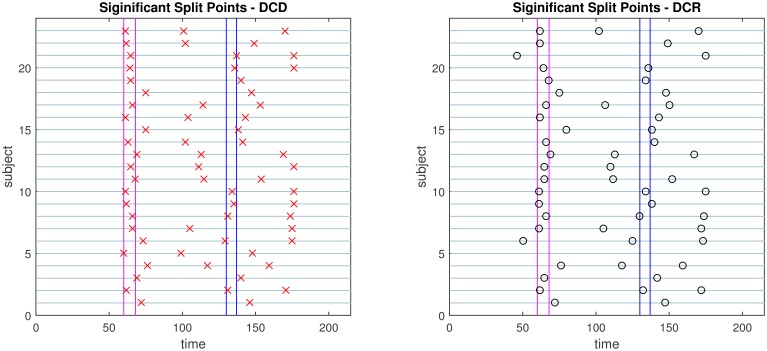
**Results of the social evaluative threat experiment, with data consisting of four ROIs and heart rate**. The *x*-axis represents time and *y*-axis depicts the subject number. The vertical lines represent the timing of the instruction slides. **Left:** Red crosses show the change points identified by DCD. **Right:** The black circles show the change points obtained via DCR.

The change points detected by the two algorithms are displayed in Figure 18. Both consistently give rise to change points around the time of the first visual cue. In addition, there appear to be changes toward the middle of speech preparation and around the time of the second visual cue, though these are less consistent across subjects. Interestingly, in contrast to the DCR algorithm, the first change points given by the DCD algorithm appears to coincide more closely to the timing of the first introduction cue. Otherwise the number, and placement, of the detected change points are roughly equivalent across methods.

### 4.2. Human connectome project

To study DCD's performance on high dimensional data, we applied the method to resting-state fMRI (rfMRI) data from the 2014 Human Connectome Project (HCP) data release (Van Essen et al., 2013). The data consists of 4 separate 15 min rfMRI runs, each consisting of 1200 time points, collected for each of 468 subjects. Each run was minimally preprocessed according to the procedure outlined in Glasser et al. (2013), with artifacts removed using FIX (FMRIB's ICA-based Xnoiseifier) (Griffanti et al., 2014; Salimi-Khorshidi et al., 2014). Each data set was temporally demeaned with variance normalization applied according to Beckmann and Smith (2004). Group-PCA output was generated by applying MELODICs Incremental Group-PCA on the 468 subjects. This comprises the top 4500 weighted spatial eigenvectors from a group-averaged PCA. The output was fed into group-ICA using FSL's MELODIC tool (Beckmann and Smith, 2004), applying spatial-ICA with 100 distinct ICA components. The set of ICA spatial maps were mapped onto each subject's time series data to obtain a single representative time series per ICA component using the “dual-regression” approach, in which the full set of ICA maps are used as spatial regressors against the full data (Filippini et al., 2009).

**Figure 19:**
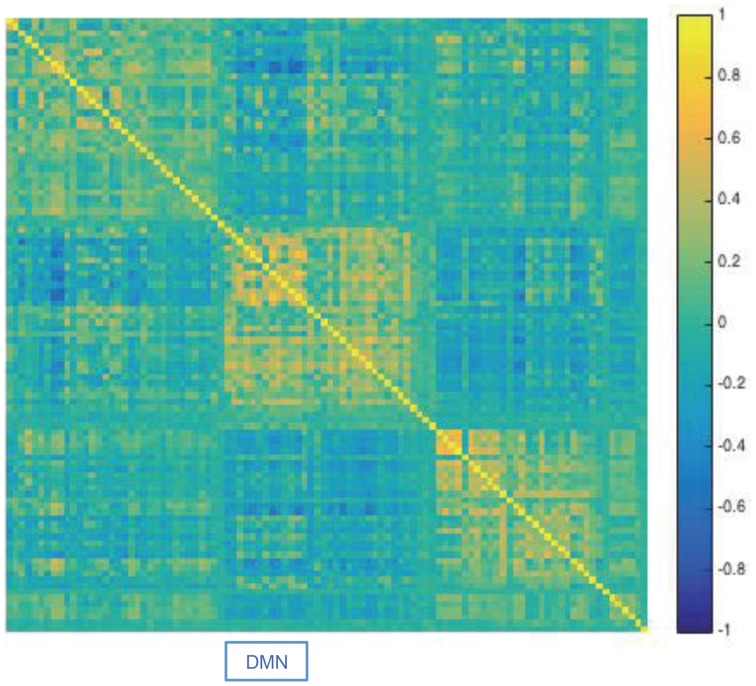
**Results of the analysis of the HCP data**. The static correlation matrix for a single subject (100307), computed using data from the four runs. Components corresponding to the default mode network are highlighted by DMN.

For illustration purposes we applied DCD to data consisting of 100 ICA component time courses from a single subject (100307). We began by computing the static correlation matrix for the subject by concatenating data across the four runs. The resulting correlation matrix was sorted using the Louvain algorithm (Blondel et al., 2008), which has proven efficient at identifying communities in large networks. The resulting correlation matrix can be seen in Figure 19. There are clear groupings of similar components that correspond to common networks seen in the resting-state literature, including the visual, somatomotor, cognitive control, and default mode networks.

**Figure 20:**
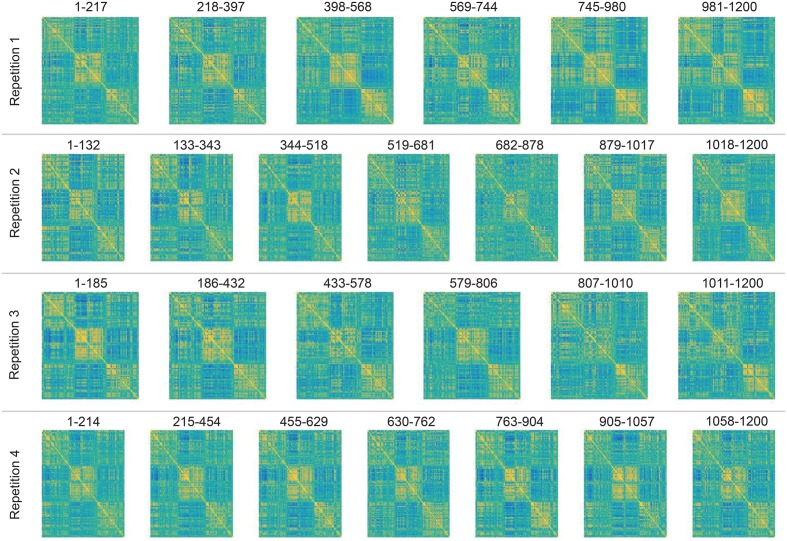
**Results of the analysis of the HCP data using DCD**. Each row depicts the estimated correlation matrices for the time partitions detected by DCD for each of 4 runs for subject 100307. Above each matrix is the temporal information for each time partition.

Next, we applied DCD with input parameters (α, β, η) = (0.05, 0.05, 0.02) to each of the four runs. The runtime for each was less than 10 s. The correlation matrices for all partitions are displayed in Figure 20, along with the corresponding temporal partition listed above them. Each run consisted of either 6 or 7 partitions, and there are clear similarities in connectivity states between runs. Here one would not expect the timing of the change points to be similar across runs, as there is no explicit task designed to invoke state changes. Rather, this example is primarily meant to illustrate that DCD is able to detect change points in situations where there are 100 nodes.

That said, these results are consistent with results seen in previous literature (Allen et al., 2014), and suggest that dynamic behavior of functional connectivity is present in the resting state data. In particular states appear to be differentiated by connectivity between default mode components, and between default mode components and other components throughout the brain.

## 5. Discussion

In this work, we have developed a novel algorithm for change point detection in fMRI data. It partitions the fMRI time series into sequences based upon functional connectivity changes between ROIs or voxels, as well as mean activation changes. DCD can be applied to time series data from ROI studies, or to temporal components obtained from either a principal components or independent components analysis. Its data-driven design means it does not require any prior knowledge of the nature of the experiment. In addition, the accuracy of the result on single subject data allows for analysis on experiments where one expects large heterogeneity in connectivity across subjects and between runs, such as in resting state fMRI data.

To reduce the burden on users, all three input parameters to the DCD algorithm have a clear statistical interpretation, making it easy to use even for those unfamiliar with the intrinsic details of the algorithm. As long as the user has a basic understanding of hypothesis testing, they should have the appropriate knowledge necessary to alter the parameters in order to improve the performance of the algorithm.

We contrast the approach to the previously introduced DCR technique, which also seeks to find connectivity change points. The most significant advantage of DCD compared to DCR is its computational efficiency, driven in large part by the newly proposed adaptive thresholding schema for sparse covariance matrix estimation. Based on the results of two high-dimensional simulation studies, as well as further empirical studies, we found that the computation time for DCR grows rapidly with an increased number of ROIs. Thus, when the number of regions exceeds 50, the computational burden of DCR can be intimidating for most users. In contrast, the computation time of DCD increases roughly linearly, and can easily handle hundreds of ROIs, in a matter of minutes for most general fMRI experimental settings.

In the DCD algorithm, we choose to maximize the total likelihood function instead of the Bayesian information criterion (BIC) that is used in the DCR algorithm. The design of the DCD algorithm frees the user from performing model selection from a list of regularization parameters, so that we can use the likelihood function as a more natural criterion. Furthermore, utilizing the likelihood function avoids a common problem arising when applying the BIC; namely that when adding the BIC score of two subsets of lengths *n*_1_ and *n*_2_ (*n*_1_ + *n*_2_ = *n*), consisting of roughly the same number of parameters *k*_1_ ≈ *k*_2_ ≈ *k*, the total penalty term is *klog*(*n*_1_)+*klog*(*n*_2_) ∝ *log*(*n*_1_(*n*−*n*_1_)), which tends to favor small or large *n*_1_ when minimizing the BIC. This is the reason for the apparent cluster of false positives obtained using DCR at time points Δ and *T* − Δ, shown in Figure 8.

Another critical difference between the two algorithms is the manner in which sparsity is enforced. DCR uses GLASSO, and thus places sparsity constraints on the precision matrix, while DCDs adaptive thresholding approach places them on the covariance matrix. The former may be more natural in the fMRI setting, due to the relationship between the precision matrix and the connectivity graph where zero elements correspond to conditional independence. However, we found in our simulation studies that when estimating connectivity change points it does not appear to be critical upon which matrix we impose sparsity, and the computational advantages of operating on the covariance matrix becomes increasingly attractive. However, in settings where the precision matrix is sparse, and the corresponding covariance matrix is dense, DCD can potentially run into problems and alternative approaches should be explored.

One limitation preventing us from further improving the runtime of the DCD algorithm comes from the nature of greedy method we used for maximizing the likelihood. The greedy search strategy makes the locally optimal choice at each step, but cannot ensure the global optimum solution is obtained. However, as a data-driven method, the results from DCD will still provide a reasonable starting point for exploring the experimental data. Another disadvantage of DCD are limits on the types of experiments it may be applied to. In this work, we have demonstrated its effectiveness using both blocked-design task fMRI experiments as well as resting state data. However, for event-related designs, the brain connectivity and activity level may change too rapidly to be able to obtain a valid estimate from DCD. Hence, when the DCD algorithm detects no significant change points, it may in fact be the case that the activity pattern changes too frequently to be detected.

Similar to group-level DCR, there is also a simple variant of DCD for group inference, which stacks subjects and calculates the summation of the likelihood function in each step. This approach can be used in experiments where one expects subjects to change states at similar time points (e.g., in the social evaluative threat experiment), and is not recommended for resting-state experiments where subjects are not expected to behave in a similar manner. In general, we suggest one first performs single-subject DCD, and if the resulting change points show synchronization across a subset of subjects, then apply group-level DCD to obtain more accurate results. Due to the flexibility of the DCD algorithm, we can also incorporate the GLASSO technique for sparse precision matrix estimation in place of adaptive thresholding method, which may also lead to improved accuracy at the cost of slower runtime.

In sum, the newly proposed DCD algorithm is a fast and efficient approach toward detecting changes in functional connectivity, especially for experiments where the nature, timing or duration of the involved psychological processes are unknown.

### Conflict of interest statement

The authors declare that the research was conducted in the absence of any commercial or financial relationships that could be construed as a potential conflict of interest.

## Appendix

### A. Minimum partition length

We need to calculate a minimum partition length Δ to control the type II error based on a pre-specified bound β. Consider two time series, each of length Δ. Denote the test statistic $t_{stat}=\frac{\delta x}{\sqrt{\frac{2}{\Delta }}s}$, where *s* represents the pooled variance. Under the null hypothesis, *t*_*stat*_ follows a Student's t-distribution with 2Δ − 2 degrees of freedom, and we reject *H*_0_ if |*t*_*stat*_| ≥ *t*_1−α/2_(2Δ − 2).

If the alternative hypothesis *H*_1_ is true, and the actual difference in mean between the two groups is δμ, then the statistic $t^{\prime }=\frac{\delta x-\delta \mu }{\sqrt{\frac{2}{\Delta }}s}$ follows a Student's t-distribution with 2Δ − 2 degrees of freedom. Without loss of generality, assume that δμ > 0. Then the type II error of this hypothesis test satisfies:

$$\begin{array}{l} Pr(|\delta x|)\leq s\cdot t_{1-\alpha /2}(2\Delta -2)\approx Pr(t^{\prime }\leq t_{1-\alpha /2}(2\Delta -2)\\ \;-\frac{\delta \mu }{\sqrt{\frac{2}{\Delta }}s})\leq \;\beta \end{array}$$

In practice, we set the effect size as $\frac{\delta \mu }{s}=1$ and since we are comparing time courses from *J* regions we use Bonferroni correction to set α → α/*J* and β → β/*J*. Beginning at Δ = 10, if $Pr\left(t^{\prime }\leq t_{1-\alpha /2M}\left(2\Delta -2\right)-\sqrt{\frac{\Delta }{2}}\right)$ is larger than β, increase Δ by 1 until the above equation is satisfied.

### B. Jackknife resampling

The jackknife is a useful technique for variance estimation. It “bootstraps” the estimator by systematically leaving out each observation and re-calculating the estimate. Suppose we have a sequence of data {*X*_*t*_}_1≤*t*≤*T*_, and we want to estimate the variance of an estimator:

$$\hat{\theta }=\frac{1}{T}\sum_{t}X_{t}$$

First we calculate the jackknife estimate of θ as

$$\theta_{Jack}=\frac{1}{T}\sum_{t}{\tilde{\theta }}_{t}$$

where ${\tilde{\theta }}_{t}$ is the estimator for a subsample omitting the *t*^*th*^ observation,

$${\tilde{\theta }}_{t}=\frac{1}{T-1}\sum_{s\neq t}X_{s}$$

Hence,

(A1) $$\begin{array}{l} \theta_{Jack}=\frac{1}{T}\sum_{t}\frac{1}{T-1}(\sum_{s=1}^{T}X_{s}-X_{t})\\ \;=\frac{1}{T-1}\sum_{t}(\frac{1}{T}\sum_{s=1}^{T}X_{s})-\frac{1}{T(T-1)}\sum_{t}X_{t}\\ \;=\frac{T}{T-1}\hat{\theta }-\frac{1}{T-1}\hat{\theta }=\hat{\theta } \end{array}$$

Now calculate an estimate of the variance of $\hat{\theta }$ using the jackknife technique:

(A2) $$\begin{array}{l} Var(\hat{\theta })=\frac{T-1}{T}\sum_{t}{\left(\tilde{\theta }-\theta_{Jack}\right)}^{2}\\ \;\;\;\;\;\;\;\;\;\;=\frac{T-1}{T}\sum_{t}{\left(\frac{1}{T-1}(\sum_{s=1}^{T}X_{s}-X_{t})-\hat{\theta }\right)}^{2}\\ \;\;\;\;\;\;\;\;\;\;=\frac{T-1}{T}\sum_{t}{\left(\frac{T}{T-1}\hat{\theta }-\frac{1}{T-1}X_{t}-\hat{\theta }\right)}^{2}\\ \;\;\;\;\;\;\;\;\;\;=\frac{T-1}{T}\sum_{t}\frac{1}{{\left(T-1\right)}^{2}}{\left(X_{t}-\hat{\theta }\right)}^{2}\\ \;\;\;\;\;\;\;\;\;\;=\frac{1}{(T-1)T}\sum_{t=1}^{T}{\left(X_{t}-\hat{\theta }\right)}^{2} \end{array}$$

Applying the result to Equation (1), we can estimate the variance of ${\hat{\Sigma }}_{ij}$ as

$$Var({\hat{\Sigma }}_{ij})=\frac{1}{(T-1)T}\sum_{1\leq t\leq T}{\left(X_{t}^{ij}-{\hat{\Sigma }}_{ij}\right)}^{2}\approx \frac{1}{T}\delta_{ij}^{2}$$

which is similar to that obtained using the central limit theorem.

### C. Simulation setting

Below is a more detailed list of simulation studies, including the exact value of precision matrices used in simulation 2–6.

- **Simulation 1**
  *Description:* The data is white noise with no connectivity change points.
  *Size: N* = 20, *T* = 1000, *p* = 20
- **Simulation 2**
  *Description:* There are two change points at times 200 and 400. Spikes are imposed onto the time series, imitating a common artifact found in fMRI data. For each subject there are 5 randomly placed spikes, each with magnitude 15.
  *Size: N* = 20, *T* = 1000, *p* = 20
  *Dependency Structure:*
  $$\begin{array}{l} \;t\in [1,200]:\;(3,14)=0.3,(3,9)=0.6,(9.14)=0.4\\ t\in (200,400]:\;(1,6)=0.7,(6,14)=0.5,(1,19)=0.6\\ t\in (400,600]:\;(3,10)=0.7,(3,13)=0.6,(3,20)=0.4,\\ \;(10,20)=0.1,(13,20)=0.1 \end{array}$$
- **Simulation 3**
  *Description:* There are three change points at times 125, 500, and 750.
  *Size: N* = 15, *T* = 1000, *p* = 20
  *Dependency Structure:*
  $$\begin{array}{l} \;t\in [1,125]:\;(2,8)=0.7,(8,17)=0.5,(2,17)=0.2\\ \;t\in (125,500]:\;(6,14)=0.1,(1,6)=0.3,(1,18)=0.2,\\ \;(1,14)=0.3,(6,18)=0.4\\ \;t\in (500,750]:\;(3,8)=0.5,(8,13)=0.5,(13,19)=\\ \;0.4,(3,19)=0.4,(3,13)=0.1,(8,19)=0.2\\ t\in (750,1000]:\;(5,11)=0.8 \end{array}$$
- **Simulation 4**
  *Description:* There is a single change point at time 100.
  *Size: N* = 25, *T* = 200, *p* = 5
  *Dependency Structure:*
  $$\begin{array}{l} \;t\in [1,100]:\;(1,3)=0.7,(3,5)=0.6,(1,5)=0.3,(3,4)\\ \;=0.2,(4,5)=0.2,(1,4)=0.1\\ t\in (100,200]:\;(1,2)=-0.1,(1,5)=-0.2,(2,5)=0.4 \end{array}$$
- **Simulation 5**
  *Description:* There are five change points at times 200, 300, 500, 600, and 800.
  *Size: N* = 20, *T* = 1000, *p* = 20
  *Dependency Structure:*
  $$\begin{array}{l} \;t\in [1,200]:\;(2,14)=0.8\\ \;t\in (200,300]:\;(2,14)=0.4,(3,9)=0.3,(9,18)=0.4,\\ \;(3,18)=0.3\\ \;t\in (300,500]:\;(3,9)=0.7,(3,18)=0.5,(9,18)=0.3\\ \;t\in (500,600]:\;(2,19)=0.4,(3,18)=0.3,(2,13)=\\ \;0.5,(6,13)=0.2,(9,18)=0.3\\ \;t\in (600,800]:\;(2,6)=0.6,(6,19)=0.5,(2,19)=0.3,\\ \;(2,13)=0.5\\ t\in (800,1000]:\;(1,11)=0.9 \end{array}$$
- **Simulation 6**
  *Description:* There are four change points at times 200, 400, 600, and 800.
  *Size: N* = 20, *T* = 1000, *p* = 20
  *Dependency Structure:*
  $$\begin{array}{l} \;t\in [1,200]:\;(1,5)=0.8,(5,10)=0.3,(10,15)=0.5\\ \;t\in (200,400]:\;(2,9)=0.6,(9,18)=0.3\\ \;t\in (400,600]:\;(3,6)=0.4,(6,13)=0.3,(13,19)=0.2\\ \;t\in (600,800]:\;(4,8)=0.7,(8,15)=0.3,(15,20)=0.6\\ t\in (800,1000]:\;(2,14)=0.5 \end{array}$$
